# Evaluation of the influence of chamomile vaginal gel on dyspareunia and sexual satisfaction in postmenopausal women: A randomized, double-blind, controlled clinical trial 

**Published:** 2020

**Authors:** Zahra Bosak, Mina Iravani, Eskandar Moghimipour, Mohammad hosein Haghighizadeh, Parivash Jelodarian, Mohammad Reza Khazdair

**Affiliations:** 1 *Student Research Committee, School of Nursing and Midwifery, Menopause Andropause Research Center, Ahvaz Jundishapur University of Medical Sciences, Ahvaz, Iran*; 2 *Department of Midwifery, Menopause Andropause Research Center, School of Nursing and Midwifery, Ahvaz Jundishapur University of Medical Sciences, Ahvaz, Iran*; 3 *Department of Pharmaceutics, School of Pharmacy, Medicinal Plant Research Center, Ahvaz Jundishapur University of Medical Sciences, Ahvaz, Iran*; 4 *Department of Biostatistics, School of Public Health, Ahvaz Jundishapur University of Medical Sciences, Ahvaz, Iran*; 5 *Department of Obstetrics and Gynecology, Fertility Infertility and Perinatology Research Center, School of Medicine, Ahvaz Jundishapur University of Medical Sciences, Ahvaz, Iran*; 6 *Cardiovascular Diseases Research Center, Birjand University of Medical Sciences, Birjand, Iran*

**Keywords:** Sexual satisfaction, Chamomile, Dyspareunia, Menopause

## Abstract

**Objective:**

The purpose of this study was to determine the effect of chamomile vaginal gel on dyspareunia and sexual satisfaction in postmenopausal women. The phytoestrogenic properties of Matricaria chamomilla were the reason for selection of this plant.

**Materials and Methods:**

This double-blind clinical trial research was conducted on 96 eligible postmenopausal women referring to Gotvand city Health Center No. 1 in 2018. In this research, 96 postmenopausal women complaining from dyspareunia and sexual dissatisfaction were randomly assigned into three groups (each contained 32 subjects) to receive 5% chamomile vaginal gel, conjugated estrogen vaginal cream and placebo gel, for 12 weeks. All women completed the Larsson and a four-degree pain self-assessment questionnaires. Data was analyzed using SPSS version 22. A p-value of less than 0.05 was considered significant.

**Results:**

After the intervention period, a significant difference was seen between the intervention and the placebo group in the mean sexual satisfaction (p<0.001). Also, a significant reduction was seen in painful sexual intercourse between the groups using vaginal gel of chamomile and conjugated estrogen vaginal cream (95% CI: chamomile: 0.68-1.04, estrogen: 0.63-0.98, placebo: 1.8-2.1; p<0.001).

**Conclusion:**

Using chamomile vaginal gel can cause a reduction in painful sexual intercourse and an increase in sexual satisfaction in postmenopausal women.

## Introduction

Menopause has been defined as a menstrual stop for one year following absence of the ovarian follicles function and reduction in estrogen (Nappi and Kokot- Kierepa, 2010 ▶). The main consequences of menopause depend mainly on estrogen deficiency (Costantino and Guaraldi, 2008 ▶). Based on epidemiological studies, around 65 to 85% of women experience unpleasant menopausal symptoms throughout their life (Berck and Novak, 2012 ▶). Vaginal atrophy is considered one of the most important factors affecting the sexual function and health of the reproductive system (Sturdee and Panay, 2010 ▶), occurring due to estrogen deficiency in postmenopausal women (Chism, 2012 ▶; Cotreau et al., 2007 ▶; Morali et al., 2006 ▶). A reduction in circulating estrogen has negative effects on elasticity and synthesis of collagen in the vulvovaginal tissue and affects the growth of the epithelial cells of the vagina. It finally causes vaginal atrophy in postmenopausal women (Sturdee and Panay, 2010 ▶). Urinary and vaginal symptoms of atrophy include vaginal dryness, pain and discomfort during sexual intercourse, bleeding after sexual intercourse, burning and urgent urination (Le Donne et al., 2011 ▶). Almost 75% of menopausal women suffer from vaginal atrophy (Panjari and Davis, 2011 ▶). One of the characteristic symptoms of vaginal atrophy is vaginal dryness, which causes painful sexual intercourse (Leiblum et al., 1983 ▶). The findings of a survey revealed that pain during sexual intercourse following vulvovaginal atrophy, leaves negative impact on the relationship of couples (Simon et al., 2014 ▶). Results of a study conducted on 3046 postmenopausal women in the United States with symptoms of vaginal atrophy, revealed that 59% of women suffered from reduced sexual satisfaction (Kingsberg et al., 2013 ▶). In a study carried out in Iran, quality of life in sexual dimension was desirable only in 45.75% of postmenopausal women (Iraji, 2005 ▶). Satisfaction from sex is a major indicator of life satisfaction (Litzinger and Gordon, 2005 ▶).

In general, the therapeutic methods used to improve the sexual satisfaction during menopause, are categorized into two groups. The first group includes the alternative hormone therapy and the second group includes complementary and alternative therapies (Warren et al., 2002 ▶). Hormone therapy is widely used, topically or systemically, to treat the effects of menopause on the genitourinary tract (Sturdee and Panay, 2010 ▶; Bachmann and Nevadunsky, 2000 ▶). Using topical estrogen improves sexual satisfaction and mitigates the dyspareunia (Nappi et al., 2013 ▶). Complementary and alternative medicine is a therapeutic method adopted increasingly among women (Taavoni et al., 2014 ▶).

Herbal treatment has special status among alternative and non-hormonal treatments, and among the herbs, the herbs containing phytoestrogen have been recommended to treat menopausal symptoms (Boroomandfar et al., 2007 ▶). Phytoestrogens can be divided into three main groups: 1) Flavonoids, 2) Coumestans and 3) Lignans, which have the ability to bind the estrogen receptors alpha and beta (Kuiper et al., 1998 ▶).

Chamomile is most commonly used for medicinal purposes (Astin et al., 2000 ▶). It is an herb of Compositae or Astraceae family. Chamomile is widely known by two types of *Matricaria chamomilla* and chamomileum nobile (Hansen and Christensen, 2009 ▶). Chamomile is used as an anti-inflammatory, antioxidant, mild sedative, anti-spasm, pain relieving herb. It is also used for the treatment of digestive problems and skin and eye diseases (Srivastava et al., 2010 ▶). The chemical components of chamomile are as follows: apigenin, apigenin-7-o-glycoside, caffeic acid, chlorogenic acid, luteolin, luteolin-7-o-glycoside, chamazolen, flavonoids (including apigenin, crocetin, pateolin and luteolin) and coumarin (Gosztola et al., 2010 ▶). Flavonoids are chemical phenyl-benzopyrones that are commonly combined with sugars and found in vascular plants. One of the six major subgroups of flavonoids is the flavone subgroup that includes flavon, apigenin, and luteolin, all present in chamomile (Brueggemeier et al., 2001 ▶; Medina et al., 1989 ▶).

 Several pharmacological studies have indicated that chamomile extract contains apigenin and chrysin flavonoids with phytoestrogenic effects capable of binding both classical and non-classical estrogen receptors and cause estrogenic effects (Sentkowska et al., 2015 ▶; Brrinhot et al., 2000 ▶; Kuiper et al., 1998 ▶). The effect of chamomile extract on bone estrogen receptors has been shown to stimulate bone cell differentiation and prevent osteoporosis (Kassi et al., 2004 ▶). The estrogenic effects of chamomile on rat testis also reduced spermatogenesis (Karbalay-Doust et al., 2010 ▶). Additionally, chamomile flavonoid compounds, through their estrogenic effects, increased motor activity in female rats (Raei et al., 2006 ▶).

There is a high prevalence of sexual problems in postmenopausal women and the lack of adequate research on sexual problems of postmenopausal women. Also, there is scanty research on the therapeutic effect of chamomile and almost no study on the impact of chamomile herb on the treatment of vaginal atrophy and its associated symptoms. Thus, the present work aimed to study the effect of chamomile vaginal gel to improve sexual problems of women during menopause and provide an appropriate solution in this context.

## Materials and Methods

This double-blind randomized controlled clinical trial recruited 96 eligible menopausal women who referred to Gotvand Health Center No. 1 in 2018. The sampling process began after approval of the project by the Ethics Committee of Ahwaz University of Medical Sciences (Committee Code: IR.AJUMS.REC.1396.729) and registration of the title in the Iranian Center for Clinical Trials (IRCT20171218037943N1).

**Study participants**

The research inclusion criteria included women who have spent their last menstruation at least one year before and have a hormonal test with 40<FSH mIU/ml, vaginal atrophy symptoms (vaginal dryness and paleness and painful intercourse), age of 45-65 years, having sexual intercourse, being monogamous and suffering from dyspareunia. Exclusion criteria included vaginal infection requiring treatment, genital tract abnormalities, hormonal therapy or using sexual hormones 8 weeks before the study, use of vaginal drugs or any lubricant at least 15 days before the study, use of alcohol, cigarette, and hubble-bubble, unspecified breast diseases, uterine bleeding with unspecified cause, consumption of high levels of phytoestrogens such as soy, red clover, fenugreek over the past month, body mass index of more than 30, liver cholestasis disorders, severe kidney failure, acute thromboembolism, or hypertension.

**Preparation of drug and placebo**

Drug and placebo were prepared by a pharmacist from Pharmacy Faculty Lab of Ahwaz. Accordingly, ground chamomile flower was extracted using 70% ethanol for 72 hr. The vaginal herbal gel was prepared by mixing the dissolved herbal extract with a suitable carrier at a concentration of 5%. Placebo gel had the same appearance as the drug and was prepared under proper conditions. Vaginal estrogen creams were prepared by Aburaihan Biruni Pharmaceutical Company. Drugs were placed in similar packages. Drugs and placebo were coded by the pharmacist.

**Methods**

Sample size was calculated as 26 people for each group based on sample size estimation after comparing the mean scores in three independent communities with a power of 90% and with a 95% confidence coefficient. With a 20% probability of dropout, 32 people were considered for each group. To choose the research samples, the researcher referred to Health Center No. 1 in Gotvand city. Then, he explained the research objectives and the way of conducting the study for those who were eligible to complete the questionnaires. After obtaining their written consent, he ensured confidentiality. Researcher and participants of research were completely unaware whether vaginal drugs contained drug or placebo. The subjects of research were assigned to the three groups of chamomile, estrogen and placebo by randomization.

Each person received one 50-gr tube along with an applicator and a brochure indicating drug use. They used it for 12 weeks, the first two weeks they used one gram every night, then in the next 10 weeks, one gram twice a week. Participants had intercourse while using the drug. Duration of use and the referral times were explained to the patient and they were asked to refer for re-examination follow up 2, 6 and 12 weeks after the onset of treatment (Suwanvesh et al., 2016 ▶; Hosseinzadeh et al., 2015 ▶; Freedman et al., 2009 ▶). The phone numbers of the subjects were taken and the way of using and follow-up times were reminded. It was also emphasized that the subjects should not use other hormonal or vaginal compounds during this time. Drug storage conditions recommended to participants were as follows: Keep the medicine in a cool, dry place, away from heat and direct light, protect from frost, and close tightly after each use. After 12 weeks of treatment, they re-completed the questionnaires and the data was analyzed.

**Data collection tools**

Personal, economic and social information of the subjects were recorded in the personal information questionnaire. The data was recorded in the questionnaire in a self-reporting manner. Severity of dyspareunia was measured by a 4-point Likert scale (0=no, 1=mild, 2=moderate, and 3=severe). The Larsson sexual satisfaction questionnaire was used to determine sexual satisfaction of the subjects. The questionnaire included 25 questions, scored in a five-point Likert scale ranging from 1 to 5. The options never, rarely, sometimes, often, and always received scores 1 to 5 in the items1-2-3-10-12-13-16-17-19-21-22-23, while these options were scored reversely in items 4-5-6-7-8-9-11-14-15-18-20-24-25. The results were categorized in this way: scores between 25 and 50 represent sexual dissatisfaction, scores between 51 and 75 represent low sexual satisfaction, scores between 76 and 100 represent moderate sexual satisfaction, and scores between 101 and 125 represent high sexual satisfaction. Validity and reliability of the original and Persian version of this questionnaire were respectively confirmed by Larson et al. and Bahrami et al. (Bahrami et al., 2016 ▶; Larson et al., 1998 ▶).

**Statistical analysis**

Data was analyzed using SPSS version 22. Chi-Square, Kruskal-Wallis, Friedman, One-Way ANOVA and Tukey HSD were also run for comparing the differences among the groups. A p-value less than 0.05 was considered of significance.

## Results

**Figure 1:**
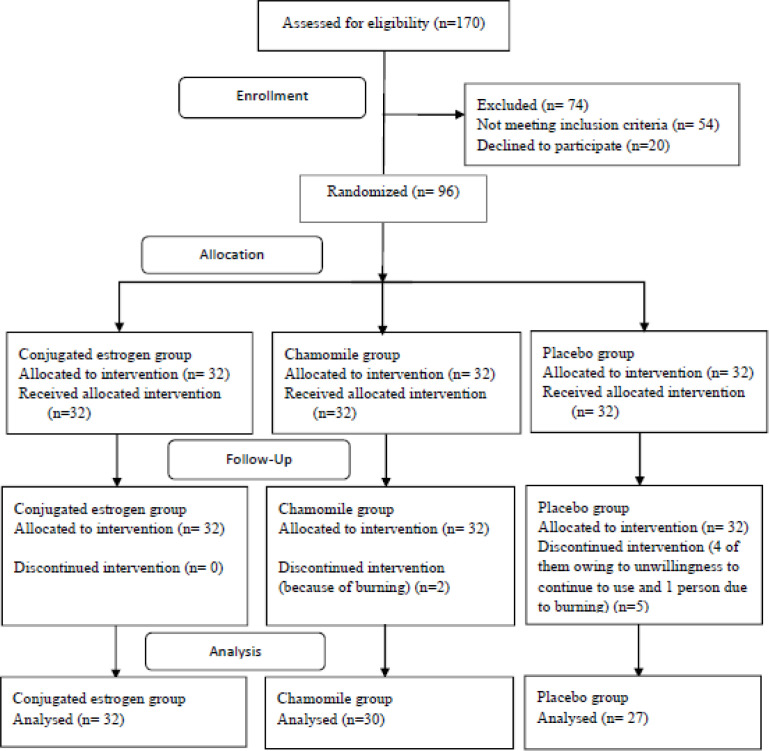
Flow chart of the study

A total of 96 postmenopausal women participated in this research, but five participants in the placebo group were excluded from research owing to unwillingness to continue to use the drugs, one in the placebo was excluded due to burning in the vagina, and two of them were excluded in the chamomile group due to burning (Figure 1). 

The average age in the chamomile, estrogen and placebo group were 53±3.4, 53.8±3.2, and 53.7±2 years respectively. Moreover, the mean age of menopause were 48.8±1.5, 49.3±1.2 and 49.5±1.3 years in the chamomile, estrogen, and placebo group, respectively, and no significant difference was found among the three groups. The highest frequency of education was elementary level of education in the three groups, including 24 subjects (80%) in the chamomile, 24 (75%) in estrogen, and 21 (77.8%) in the placebo group. The most frequent number of sexual activity was once per week in treated groups (20 (66.7%) in the chamomile group, 19 (59.4%) in the estrogen group, and 16 (59.3%) in the placebo group). The economic status was in moderate level for 21 people (70%) in chamomile group, 23 (71.9%) in estrogen group, and 20 (74.1%) in the placebo group. In addition, 29 (96.7%) in the chamomile group, 30 (93.8%) in the estrogen group, and 26 (96.3%) in the placebo group, were housewives (Table 1). In this research, the three groups did not show significant differences in terms of socio-demographic characteristics (p>0.503) (Table 1). 

Based on the Kruskal-Wallis test, dyspareunia severity before and after intervention in chamomile and conjugated estrogen groups was significantly different from that of placebo group (p<0.001), but there was no significant difference between chamomile group and conjugated estrogen groups (p<0.678). In addition, in the first visit, 9 (30%) people had severe dyspareunia, 16 (53.3%) had moderate dyspareunia and 5 people (16.7%) had mild dyspareunia in chamomile group. In the second visit, 9 (30%) people had moderate dyspareunia, 12 (40%) people had mild dyspareunia, and 9 (30%) had no dyspareunia in the chamomile group, and this difference between chamomile and placebo groups was statistically significant (p<0.001). At the end of the intervention, 29 (96.7%) had no dyspareunia and 1 (3.3%) person expressed mild dyspareunia in the chamomile group, indicating a positive effect for chamomile vaginal gel. Based on the results, dyspareunia recovery showed no significant difference in chamomile and conjugated estrogen groups, but showed significant difference between treatment and the placebo groups (95% confidence interval: chamomile:0.68-1.04, estrogen: 0.63-0.98, and placebo:1.8-2.1; p=0.001). Recovery in dyspareunia symptoms, since the second week of treatment, in the chamomile group was similar to that of the conjugated estrogen group (Table 2).

The average sexual satisfaction score in subjects treated with chamomile vaginal gel was 66.8±16.3 before intervention and 88.4±14.9 after intervention, and the mean score in the group that used conjugated estrogen vaginal was 65.5±16.3 before the intervention and 88.7±12.9 after the intervention (Table 3). The groups were compared in pairs using Tukey HSD test. Based on this test, significant difference was not found between the chamomile group and the conjugated estrogen group, (Table 4). Compared to before-intervention, the sexual satisfaction score increased significantly in the chamomile group, which was similar to that of the conjugated estrogen group. There was a significant difference between the chamomile and placebo groups as well as the estrogen group with placebo.

**Table 1:** Personal characteristics of subjects (n=96)

**Personal characteristics**		**Chamomile**	**Conjugated estrogen**	**Placebo**	**p-value**
Age (year)		53±3.4	53.8±3.2	53.7± 2	0.503
Menopausal age (year)		48/8±1.5	49/3±1/2	49/5±1/3	0.121
Menarche age (year)		14.2±1.1	14.3±1.2	13.8±1.8	0.445
BMI		24.5±0.29	24.6±0.24	24.4±0.42	0.194
Education	Elementary	24(80%)	24 (75%)	21 (77.8%)	0.958
	secondary	4 (13.3%)	6 (18.8%)	5 (18.5%)	
	High school	2 (6.7%)	2 (6.3%)	1 (3.7%)	
	Academic	0	0	0	
job	employed	1 (3.3%)	2 (6.3%)	1 (3.7%)	0.834
	housewife	29 (96.7%)	30 (93.8%)	26 (96.3%)	
	Retired	0	0	0	
Economic status	Poor	5 (16.7%)	6 (18.8%)	5 (18.5%)	0.963
	Moderate	21 (70%)	23 (71.9%)	20 (74.1%)	
	Excellent	4 (13.3%)	3 (9.4%)	2 (7.4%)	
Number of intercourse per week	0	2 (6.7%)	3 (9.4%)	3 (11.1%)	0.993
	1	20 (66.7%)	19 (59.4%)	16 (59.3%)	
	2	4 (13.3%)	6 (18.8%)	5 (18.5%)	
	3	1 (3.3%)	2 (6.3%)	1 (3.7%)	
	4	3 (10%)	2 (6.3%)	2 (7.4%)	

Data is presented as mean±SD and number (percent). Statistical analyses were done using the one-way ANOVA and Kruskal- wallis test.

**Table 2:** Comparison of dyspareunia severity before intervention and at weeks 2, 6, and 12 after the study

**Time**		**Chamomile**	**Conjugated estrogen**	**Placebo**	**p-value** *
Before treatment	no	0	0	0	0.870
	mild	5 (16.7%)	5 (15.6%)	4 (14.8%)	
	moderate	16 (53.3%)	17 (53.1%)	15 (55.6%)	
	severe	9 (30%)	10 (31.3%)	8 (29.6%)	
Week 2	no	9 (30%)	11 (34.4%)	0	0.001
	mild	12 (40%)	15 (46.9%)	5 (18.5%)	
	moderate	9 (30%)	6 (18.8%)	17 (63%)	
	severe	0	0	5 (18.5%)	
Week 6	no	21 (70%)	25 (78.1%)	0	0.001
	mild	9 (30%)	7 (21.9%)	7 (25.9%)	
	moderate	0	0	15 (55.6%)	
	severe	0	0	5 (18.5%)	
Week 12	no	29 (96.7%)	31 (96.9%)	0	0.001
	mild	1 (3.3%)	1 (3.1%)	8 (29.6%)	
	moderate	0	0	14 (51.9%)	
	severe	0	0	5 (18.5%)	
p-value**		0.001	0.001	0.002	

Data is presented as number (percent).

P-value*: comparison of the three groups at the beginning of the study and after the intervention (Week 2, 6 and 12), made using kruskal Wallis test.

P-value**: Compared using Friedman test for changes within the groups.

**Table 3:** Comparison of mean scores of sexual satisfaction in groups received chamomile vaginal gel, conjugated estrogen vaginal cream, and placebo

**Sexual satisfaction score**	**Chamomile** **SD** **±** **mean**	**Conjugated estrogen** **SD** **±** **mean**	**Placebo** **SD** **±** **mean**	p-value*
Before intervention	66.8±16.3	16.3±66.5	15.7±71.8	0.300
After intervention	88.4±14.9	12.9±7.88	16.2±76.4	0.003

Data is presented as mean±SD.

* One-way ANOVA was used to compare the mean of changes between the three groups, before and after the intervention.

**Table 4:** Pairwise comparison of groups

**Group**	**group**	**Mean difference**	p-value*
Chamomile	Conjugated estrogen	1.3 ±-1.6	0.467
	Placebo	17±1.4	0.001
Conjugated estrogen	Chamomile	1.3 ±1.6	0.467
	Placebo	18.6±1.4	0.001
Placebo	Chamomile	-17±1.4	0.001
	Conjugated estrogen	-18.6±1.4	0.001

Data is presented as mean±SD. * The comparison of groups was made by Tukey’s test *post hoc*.

## Discussion

In the current research that was conducted with the aim of evaluating the effect of chamomile vaginal gel on dyspareunia and level of sexual satisfaction in postmenopausal women, the use of chamomile vaginal gel similar to conjugated estrogen vaginal cream significantly positive effect in reducing dyspareunia and increasing sexual satisfaction in postmenopausal women.

By reducing the blood supply to the genital tract and vagina, reduction or inhibition of the release of estrogen and androgen hormones during menopause, physiologically atrophy is caused in these areas, and it is accompanied by symptoms such as dryness, paleness and dyspareunia. It is also followed by painful intercourse and reduced sexual desire (Brizendine, 2004 ▶). 

In a randomized, double-blind study conducted by Archer et al. (2018) ▶ to assess the efficacy and safety of 0.003% vaginal estradiol cream in postmenopausal women with vaginal dryness, 576 postmenopausal women with vaginal dryness symptoms were enrolled and the results showed that the use of conjugated vaginal estrogen cream reduced the severity of vaginal dryness and reduced dyspareunia after 12 weeks, compared to placebo. Also, in the study conducted by Eftekhar et al. (2009) ▶ to investigate the effect of vaginal estrogen on mood and sleep disorders and sexual satisfaction in postmenopausal women, the rate of sexual satisfaction in the conjugated vaginal estrogen cream group was significantly higher than placebo. The findings of the two mentioned studies indicated that vaginal estrogen cream reduced dyspareunia and increased sexual satisfaction, which is consistent with the findings of the present study.

Review of studies indicated that no study has been conducted on the effect of chamomile vaginal gel on sexual activity in postmenopausal women, but clinical trials have shown positive effects of herbs containing phytoestrogens in reducing the menopausal symptoms severity. Studies have also indicated the effects of chamomile on improving some menopausal symptoms. 

Consistent with the results of our study, Taavoni et al. (2014) ▶ in a triple-blind clinical trial showed that Aphrodite herbal supplement containing ginger, cinnamon, saffron and truffle, improved the sexual satisfaction in postmenopausal women after one month, compared to a placebo group. Also, Morali et al. (2006) ▶ conducted a research to determine the effectiveness and safety of therapeutic methods as topical and intra-vaginal gel in postmenopausal women with vaginal atrophy. In this uncontrolled clinical trial, patients used vaginal gel containing sodium salt of hyaluronic acid, phytoestrogens derived from extract of common hop, liposomes and vitamin E. Results showed a significant decrease in the severity of dyspareunia only in the first week of treatment, so that vaginal symptoms were reduced in the final visit (12 weeks after treatment). Their results are in line with those of our study. In a research conducted by Mazalzadeh et al. (2018) ▶ to examine the effect of fenugreek seed vaginal cream on dyspareunia and sexual satisfaction in postmenopausal women, it was revealed that the use of fenugreek seed vaginal cream for 8 weeks mitigated intercourse pain and increased sexual satisfaction in postmenopausal women compared to placebo. Their results were consistent with those of the present study.

As we mentioned at the beginning of the discussion, the chamomile vaginal gel decreased dyspareunia and improved sexual satisfaction. This effect might be possibly due to the presence of phytoestrogens. Numerous studies on plants have confirmed the function of phytoestrogens as estrogens (Karbalay-Doust et al., 2010 ▶; Raei et al., 2006 ▶; Kassi et al., 2004 ▶). Studies have revealed that the phytoestrogens found in herbal extracts, are capable to bind estrogen receptors (Jarry et al., 2003 ▶). Thus, as estrogen level is expected to be low in postmenopausal women, phytoestrogens to apply their estrogenic effects more strongly, and to reduce some menopausal symptoms (Azadbakht, 2007 ▶). Phytoestrogens in the herbal extracts of chamomile flower act similarly as sexual steroids in the females (Perrot-Sinal et al., 2000 ▶). Several studies have been conducted to investigate the estrogenic effects of chamomile. For example, Hatami et al. (2013) ▶ reported that the estrogenic effects of chamomile extract increased serum levels of Luteinizing hormone (LH), testosterone and pituitary-gonadal axis activity in adult mice. Also, in a research conducted by Kupfersztain et al. (2003) ▶ to investigate the efficacy of climex (a combination of chamomile extract and Angelica sinensis) for the treatment of menopausal hot flashes, a significant difference was seen between the study and control groups in terms of reducing the number and severity of hot flashes after 12 weeks of treatment. In our study, chamomile probably through its phytoestrogenic effects, with an estrogen-like mechanism, eliminated vaginal dryness and consequently, decreased dyspareunia and increased sexual satisfaction in postmenopausal women.

However, a meta-analysis conducted by Najafi et al. (2018) ▶ with the aim of examining the effects of phytoestrogens on post-menopausal female sexual dysfunction showed that phytoestrogens have different effects on sexual function. Furthermore, they all had no promising effect on the sexual function, which is contrary to the results of our study. In one-fifth of people, isoflavones is not absorbed without any specific cause (Pitkin, 2004 ▶), which may be the reason for the lack of response to treatment in some studies on the effects of isoflavones. The difference in results can also be due to differences in product type, dosage and duration of use. 

One of the strengths of the present study was investigating, for the first time, the effect of chamomile vaginal gel on dyspareunia and sexual satisfaction in postmenopausal women. In this study, the efficacy of chamomile vaginal gel was compared with both placebo and standard treatment. Also this study was a double-blind randomized controlled clinical trial. One of the limitations of this study was the lack of control over all factors affecting sexual satisfaction, including mental states in the subjects, for which the control group could not help. 

The participants reported no adverse side effects for the use of chamomile vaginal gel except for burning reported by two participants, who were excluded from the research. Thus, chamomile vaginal gel can be a good alternative to hormonal treatment.

It seems that the use of chamomile vaginal gel for 12 weeks, mitigates the intercourse pain and increases sexual satisfaction in postmenopausal women. Although no important side effect was observed in this study for the use of chamomile vaginal gel, further studies are recommended with higher accuracy and larger sample size to be conducted to evaluate the effect and safety of this drug.
